# Lessons learnt on patient safety in dentistry through a 5-year nationwide database study on iatrogenic harm

**DOI:** 10.1038/s41598-024-62107-x

**Published:** 2024-05-19

**Authors:** Nikolaos Ferlias, Henrik Nielsen, Erik Andersen, Peter Stoustrup

**Affiliations:** 1 Section of Orthodontics, Department of Dentistry and Oral Health, Aarhus University, 8000 Aarhus, Denmark; 2 Department of Oral and Maxillofacial Surgery, Copenhagen University Hospital, Copenhagen, Denmark; 3 Private Practice, Colosseum Dental Group, Broendby, Copenhagen, Denmark

**Keywords:** Dentistry, Dental treatments

## Abstract

Safe delivery of care is a priority in dentistry, while basic epidemiological knowledge of patient safety incidents is still lacking. The objectives of this study were to (1) classify patient safety incidents related to primary dental care in Denmark in the period 2016–2020 and study the distribution of different types of dental treatment categories where harm occurred, (2) clarify treatment categories leading to "nerve injury" and "tooth loss" and (3) assess the financial cost of patient-harm claims. Data from the Danish Dental Compensation Act (DDCA) database was retrieved from all filed cases from 1st January 2016 until 31st December 2020 pertaining to: (1) The reason why the patient applied for treatment-related harm compensation, (2) the event that led to the alleged harm (treatment category), (3) the type of patient-harm, and (4) the financial cost of all harm compensations. A total of 9069 claims were retrieved, of which 5079 (56%) were found eligible for compensation. The three most frequent categories leading to compensation were "Root canal treatment and post preparation"(n = 2461, 48% of all approved claims), “lack of timely diagnosis and initiation of treatment” (n = 905, 18%) and “surgery” (n = 878, 17%). Damage to the root of the tooth accounted for more than half of all approved claims (54.36%), which was most frequently a result of either parietal perforation during endodontic treatment (18.54%) or instrument fracture (18.89%). Nerve injury accounted for 16.81% of the approved claims. Total cost of all compensation payments was €16,309,310, 41.1% of which was related to surgery (€6,707,430) and 20.4% (€3,322,927) to endodontic treatment. This comprehensive analysis documents that harm permeates all aspects of dentistry, especially in endodontics and surgery. Neglect or diagnostic delays contribute to 18% of claims, indicating that harm does not solely result from direct treatment. Treatment harm inflicts considerable societal costs.

## Introduction

In the past two decades, emerging attention has been paid to the consequences of unsafe healthcare delivery and healthcare-related harm^1^. Patient safety research has transformed into a specific body of knowledge and implementation of safety measures has become a priority across all health care settings^2^. The World Health Organization (WHO) defines “patient safety” as the “freedom for a patient from unnecessary harm or potential harm related to healthcare”^3^. Furthermore, WHO defines “harm” as “impairment of structure or function of the body and/or any deleterious effect arising there from”. Safe care forms a core component to the delivery of high-quality healthcare^4^. Over the last 20 years, there has been an increasing scientific focus and recognition of patient safety incidents. However, specific patient harm in primary healthcare settings specifically related to dentistry is understudied, while efforts have been raised to improve our understanding^5,6^.

Oral diseases are among the most prevalent global diseases affecting more than 3.5 billion people worldwide^7^. In high and middle-income countries, most dental care is characterized by a specialized, highly technical, interventional approach originating from the primary healthcare setting^8^. The invasive nature of dental care, the close contact between provider and patient, and the large proportion of individuals seeking oral healthcare^9^ constitutes an obvious high-risk potential for patient-safety incidents and iatrogenic harm. Reports have elucidated the nature of patient-safety incidents and patient harm in dental care^10–12^. Across the literature, the reported findings of nature and degree of harm have varied with the groups examined, difference in taxonomy, and the underlying sources of data (e.g. type of reporting systems)^13^.

Delivery of safe care is a priority in dentistry^10^. Still, the medical professions seem to outperform dentistry in advancement of patient safety culture initiatives and education^14^. Recently, candidate lists of “never events” have been developed for primary dental care^15,16^. To enhance awareness of safety culture, a recent systematic review has highlighted the importance of implementing aspects of patient safety in the undergraduate dental curriculum^17^. Multi-professional patient safety curriculums have been developed by the WHO to enhance patient safety education^18^. However, there is a lack of evidence supporting specific actions to enhance patient safety in dentistry^19^. Basic epidemiological knowledge of patient harm related to primary dental care is still lacking^19,20^. Aspects like contributing factors, the patient perspective of burden of harm, and the financial costs and expenses related to repair and rehabilitation after patient harm, need further elucidation to inform the design of proactive interventions and policies to enhance patient safety in primary dental care^19^.

The objectives of the present study were (1) to classify patient harm related to primary dental care in the period 2016–2020 and study the distribution of types of dental treatment categories that led to patient harm, (2) to identify treatment categories leading to "nerve injury" and "tooth loss" (never events) and 3) to assess the financial cost of patient-harm claims from dental care.

## Material and methods

The current study adheres to the WHO terminology on patient safety^3^ and complies with the STROBE (Strengthening the reporting of observational studies in epidemiology) guidelines^21^.

### Ethics

All methods in the present study were conducted in accordance with the relevant guidelines, regulations and rules from the Danish Health Authorities regarding the use of data from clinical databases. All protocols in this study, storage and handling of sensitive personal data was approved by the Danish Dental Compensation Act (DDCA) prior to initiation of the study. In accordance with Danish legislations on healthcare databases, informed consent to use the data was granted by the DDCA on behalf of the included subjects in accordance with Danish regulations on the use of data from official clinical databases. The project was registered to the Danish Data Protection Agency through registration at Aarhus University, Denmark (No: 2022-0367531, no 3088).

### Data source

The current project is based on data from the DDCA, which is a scheme based on legislation. The legislation gives rights to patients who suffer physical or mental injury during dental care to apply for financial compensation. The legislation is a no-fault compensation scheme. The purpose of this scheme is to assist with the costs of treatment and rehabilitation for patients who suffer harm during dental care. The DDCA compensation does not cover illness or lack of effect from a treatment nor because the treatment did not “cure” the patient^22^.

The DDCA compensation scheme is separate from the legislation that deals with malpractice. A claim for treatment-related harm compensation is not dependent on a complaint against a practitioner. The criterion for compensation is that the injury most likely occurred as a result of the treatment and had negative aftereffects for the patient. Injuries that can be restored by redoing the treatment are not eligible for compensation by law.

All patients have the right to file a claim if they believe they have experienced harm during dental treatment. To initiate this process, the patient completes a short online form and submits it to the Danish Dental Association, which forwards the case to the DDCA. No lawyer is needed. The DDCA administrative staff then gathers and reviews all relevant documents from both the patient and the dental provider. A dental specialist evaluates the case, considering the treatment progression and expected standards of the treatment provided. Subsequently, an unbiased legal expert from the DDCA reviews this evaluation and issues a final verdict. Both parties are entitled to appeal this decision. Importantly, patients do not need legal representation at any point in this tax-funded process, which is free of charge for the patient.

The legislation covers all authorized dentists and dental hygienists in Denmark. Therefore, data from the scheme is considered representative of the patient safety incidents in Denmark. The electronic database contains detailed and standardized information retrieved (demographics, treatment procedure, type of harm, etc.) and extracted from each single claim and categorized by an expert dental professional in connection with the case review assessment process.

### Data acquisition and analysis

We undertook a retrospective collection of data from the DDCA database retrieving all filed cases from 1st January 2016 until 31st December 2020, including the financial costs of harm compensations for the same period. These costs were available two years after the claims were submitted, hence the year selection, since it takes time from the acceptance of the injury to the final decision and settlement of the rehabilitation cost. From the files, we extracted standardized data pertaining to: (1) The reason why the patient applied for treatment-related harm compensation, (2) the event that led to the alleged harm (treatment category), and (3) the type of patient-harm. The extracted data consisted of claims that were accepted and led to the payment of compensation as well as claims that were rejected as they were not considered as treatment-related harms and deemed ineligible for compensation. Specific data was extracted on claims that led to compensation for one of the two predefined never events: "nerve injury" and "tooth loss". The concept “Never events” points towards patient-safety incidents where avoidance is prioritized with the implementation of preventable measures by healthcare workers^15,16^. Descriptive statistics were applied to illustrate the relative proportion (prevalence) of events during the 5 years of observation.

## Results

The DDCA received a total of 9069 claims in the period from 1st January 2016 till the 31st December 2020. Out of the total number of notified claims (n = 9069), a total of 5079 claims were approved as treatment-related harms and found eligible for compensation (56%) during the 5-year period. Table 1 shows the types of treatment categories that led to the claims of treatment-related harm, following the DDCA categorization.

**Table 1:** Treatment categories leading to claim of treatment-related harm during dental care.

Treatment categories that led to claim of harm	Total number of notified claims alleged between 2016 and 2020	Total number of approved claims found eligible for compensation between 2016 and 2020 (the relative proportion of approved claims vs. total number of alleged claims in each treatment category)	Relative proportion of each treatment category compared to total number of approved claims (n = 5079)	Number of notified cases needed for each approved claim eligible for compensation
Root canal treatment and post preparation	3870	2461 (63.6%)	48.5%	1.6
Delayed diagnosis/treatment	1400	905 (64.6%)	17.8%	1.6
Surgery	1273	878 (70%)	17.3%	1.5
Conservative treatment^a^	1373	451 (32.9%)	8.9%	3
Anaesthesia	264	216 (81.8%)	4.3%	1.2
Implantology	374	91 (24%)	1.8%	4.1
Orthodontic treatment	231	48 (20.8%)	0.9%	4.8
Periodontal treatment	51	7 (13.7%)	0.1%	7.3
Removable prosthetics	73	5 (6.9%)	0.1%	14.6
Functional treatment	18	3 (17%)	0.1%	6
Cosmetic dentistry	13	2 (15%)	0.04%	6.5
Other	129	12 (9.3%)	0.2%	10.8
Total	9069	5079 (100%)	100%	1.8

^a^Conservative treatment involved crowns, bridges, fillings and composite build-ups.

### Treatment categories leading to harm

Table 1 contains data from the total number of notified cases (n = 9069), as well as data from the approved claims that were found eligible for compensation (n = 5079). The three most frequent categories leading to approval and payment of compensation were "Root canal treatment and post preparation" (48% of all approved claims), lack of timely diagnosis and initiation of treatment (18%) and surgery (17%). The categories with the lowest ratio between notified claims and approved claims were: "Anesthesia" (1.2 claims), "surgery" (1.5 claims), "lack of timely diagnosis" (1.6 claims) and "Root canal treatment and post preparation” (1.6 claims). On the contrary, the category with the highest ratio was “removable prosthetics” (14.6 claims).

### Types of treatment-related harms

Table 2 presents the relative distribution of harm related to the total amount of approved claims (n = 5079) during the 5 years of observation. Damage to the root of the tooth accounted for more than half of all approved claims (54.36%). In this overall category, the two most relatively frequent injuries were loss of tooth due to parietal perforation during endodontic treatment (18.54%) and instrument fracture during endodontic treatment leading to apical infection and need for more extensive treatment in the form of apical surgery (18.89%). The second most frequent harm was nerve injury which accounted for 16.81% of the approved claims, followed by damage to crown and roots due to lack of timely diagnosis and initiation of treatment (15.78% of the approved claims)**.**

**Table 2:** Types of treatment-related harms during dental care and the consequences of the harm.

Category of injury	Relative proportion (%)^a^
Damage to tooth root (54.36%^a^)	
Fractured root file—apical infection. surgical treatment indicated	18.89
Perforation—tooth loss	18.54
Perforation—repair without tooth loss	4.04
Fractured root file—loss of tooth	3.33
Other complication of root canal treatment—tooth loss	1.93
Root fracture—loss of tooth	1.18
Root resorption—tooth loss	0.74
Fractured root post—loss of tooth	0.66
Drilling injury—loss of tooth	0.56
Fractured root file—treated by removal of the root instrument	0.44
Other	4.04
Permanent nerve damage (16.81%^a^)	
Inferior alveolar nerve	6.95
Lingual nerve	5.04
Buccal nerve	1.51
Mental nerve	1.15
Multiple nerves affected	0.83
Infraorbital nerve	0.52
Facial nerve	0.05
Other nerves	0.71
Delayed diagnosis and treatment (15.78%^a^)	
Caries—loss of tooth	6.28
Caries—root weakening	3.40
Periodontitis—tooth loss	2.06
Periodontitis—loss of attachment	1.15
Caries—crown weakening	1.10
Periodontitis—weakening of teeth/function	0.15
Periodontal disease—crown weakening	0.07
Cancer	0.07
Other	1.51
Damage to dental crown (7.02%^a^)	
Loss of dental crown substance (enamel. dentin or restoration)	3.70
Pulp damage	2.08
Tooth fracture	1.23
Various (3.18% n = 162)^a^	
Treatment or removal of an incorrect tooth	1.18
Infection	0.91
Pain	0.69
Disfigurement	0.30
Loss of components (e.g. crown over implant)	0.02
Aspiration or ingestion	0.01
Damage to anatomical structure other than tooth/nerve (2.25%^a^)	
Sinus perforation	0.74
Loss of bone	0.66
Soft tissue injury	0.59
Jaw fracture	0.17
Loss of major parts of the jawbone	0.05
Major haemorrhage	0.03
Negative impact on implant (0.34%^a^)	
Loss of implant	0.22
Loss of bone	0.12
Negative impact on function (0.25%^a^)	
Functional impairment/pain	0.14
Discomfort from muscles and/or joints	0.12

^a^Relative proportion to the total number of approved harms (n = 5079) in the period from 2016 to 2020.

### Subdivision of harms related to the three most frequent harm-categories

**Figure 1:**
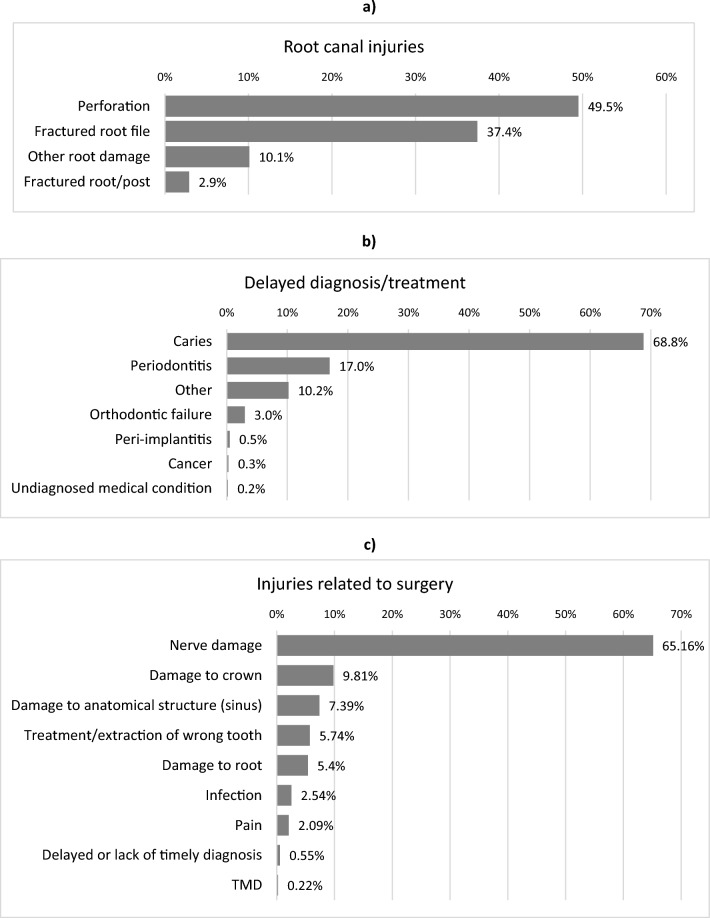
(**a**) Injuries in the “Root canal treatment and post preparation” category. Relative distribution of injuries within the category for the period 2016–2020 depicted in Table 1 (n = 2461)**.** (**b**) Delayed diagnosis and lack of initiation of timely treatment. Relative distribution of harm categories in the category "*Delayed diagnosis and treatment*" in the period 2016–2020 depicted in Table 1 (n = 905). (**c**) Injuries related to “surgery”. Relative distribution of harms related to surgical treatment in the period from 2016 to 2020 depicted in Table 1 (n = 878).

Figures 1a–c present a subdivision of the harms related to the three most frequent categories of harm (as shown in Table 1). Relative distribution of injuries within each category reveals the most common type of injury when it comes to: “root canal treatment and post preparation”, “delayed diagnosis and lack of timely treatment” and “injuries related to surgery”. Supplemental material online only presents data on the surgical treatment modalities leading to harm.

### Never event—nerve injury

**Figure 2:**
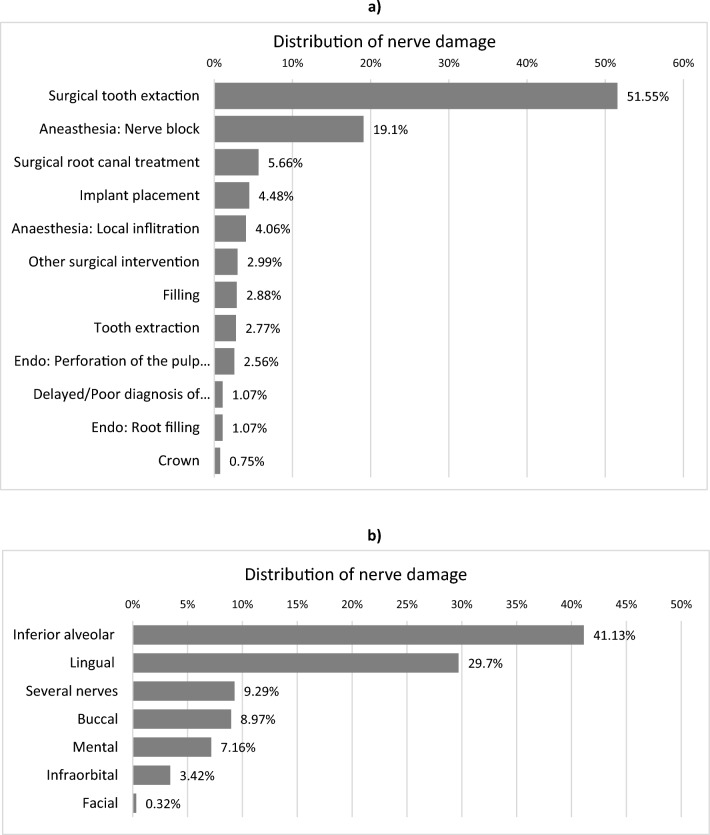
(**a**) Treatment categories leading to the never event harm “permanent nerve injury”. The relative distribution of treatment categories that led to permanent nerve damage in the period from 2016 to 2020. (**b**) Permanent nerve injuries. Relative distribution of the types of permanent nerve injuries in the period from 2016 to 2020.

From 2016 to 2020, claims from 937 permanent nerve injuries were found eligible for compensation. The treatment categories most frequently causing the permanent nerve injuries were “surgical removal of a tooth” (51.55%), the application of nerve block anesthesia (19.10%), surgical endodontic procedures (5.66%), and implant placement (4.48%) (Fig. 2a). The most common types of permanent nerve injuries involved the inferior alveolar nerve of the mandibular nerve (41%), the lingual nerve (30%), and a combination of 2 or more nerve branches (9%) (Figs. 2b).

### Never event—loss of tooth

A total of 1979 claims were approved and found eligible for compensation in the harm-category “loss of tooth”. The treatment categories most frequently causing “loss of tooth” harm were perforation of pulp chamber during cavity preparation (22.99%), root canal preparation during endodontic treatment (21.48%), neglected or delayed diagnosis/initiation of treatment leading to caries (17.74%), and insertion of post in the root canal (16.47%) (Supplementary Fig. 2).

### Financial costs of harms from 2016 to 2020

The total financial costs of compensations related to rehabilitation of harm was € 16,309,310 (Table 3). Harms related to surgical treatments constituted of 41.13% of the total costs followed by endodontic treatments (20.37%) and neglected or delayed diagnosis and initiation of treatment (13.9%). The treatment categories with the highest average compensation per approved claim were harms related to application of anesthesia (€ 9491.92 per claim) and harms related to surgical procedures (€ 6946.30). Both these categories include many of the injuries that led to permanent nerve damage, where the extent of the injury gives rise to a degree of disability that is also compensated for.

**Table 3:** Financial costs to repair harms from 2016 to 2020^a^.

Treatment category	Number of cases	Total compensation payments in the period from 2016 to 2020 in Euro	Percentage of compensation payment in relation to total payment	Avg. cost per claim in the period from 2016 to 2020 in Euros
Root canal treatment and post preparation	2387	€ 3,322,927.27	20.4	€ 1392.1
Surgery	921	€ 6,707,430.00	41.1	€ 7282.77
Neglected or delayed diagnosis and lack of timely initiation of treatment	786	€ 2,260,410.05	13.9	€ 2875.84
Conservative treatment	450	€ 1,298,342.58	8.0	€ 2885.20
Anaesthesia	224	€ 2,105,556.05	12.9	€ 9399.8
Implantology	105	€ 439,390.86	2.7	€ 4184.67
Orthodontic treatment	48	€ 80,687.01	0.5	€ 1680.98
Others	13	€ 31,775.01	0.2	€ 2444.23
Periodontal treatment	10	€ 35,340.88	0.2	€ 2444.23
Cosmetic treatment	5	€ 12,878.36	0.1	€ 2575.67
Functional treatment	4	€ 5146.82	0.03	€ 1286.71
Removable prosthetics	3	€ 7764.04	0.05	€ 2588.01
**Total**	4958^a^	€ 16,309,310.34	100.0	€ 3289.49

^a^The expenditure represents compensation for cases notified from January 1, 2016 until December 31, 2020 that are currently settled. The number of cases where compensation is currently paid amounts to 4958 claims, which is lower than the total number of approved cases from the same period (n = 5079).

## Discussion

The findings of this study contribute to significant insights into patient safety incidents and treatment-related harms in primary dental care. By analyzing a large dataset from the Danish Dental Compensation Act (DDCA) database, this study provides a comprehensive understanding of the distribution of harms, treatment categories associated with patient harm, and the financial costs of patient-harm claims. These findings have important implications for improving patient safety and enhancing the quality of dental care.

A strength of this study lies in the comprehensive analysis of a large dataset, which includes a substantial number of claims, as all of those submitted to the DDCA over a 5-year period were included. This dataset provides a robust representation of patient safety incidents in dental care, capturing a wide range of treatment-related harms. By extracting detailed and standardized information, this study enhances the accuracy and reliability of the findings, enabling a thorough examination of the specific categories of treatment associated with patient harm. The identification of specific treatment categories leading to patient harm is a crucial aspect of this study.

The results demonstrate that "Root canal treatment and post preparation," "lack of timely diagnosis and initiation of treatment," and "surgery" are the most frequent categories associated with patient harm. Notably, neglect or lack of timely diagnosis is responsible for 18% of all approved and reported claims, indicating that harm does not arise solely from an active medical act. These results come in agreement with an investigation in Finland from Hiivala et al. on patient-safety incidents in all fields of dentistry, where 16% of all incidents were related to diagnostics^11^. Additionally, a retrospective review of adverse events also reports that the largest type of harm was related to diagnostic errors^10^. The present study provides knowledge that allow dental practitioners and policymakers to focus their efforts on these areas, implementing targeted interventions and quality improvement initiatives to minimize patient harm and enhance the safety of dental care delivery.

Furthermore, the analysis of the types of harm within these categories provides valuable insights into the nature and consequences of patient harm in dental care. The study reveals that damage to the root of the tooth is the most common type of harm, accounting for over half of all approved claims. Within this category, parietal perforation during endodontic treatment and instrument fracture during endodontic treatment are identified as significant factors contributing to patient harm. Additionally, nerve injuries and the loss of teeth are prevalent types of harm, underscoring the importance of addressing these specific areas to improve patient safety^11,23^. A similar investigation in Spain from Perea-Pérez et al. reports that endodontics is the second most common dental procedure related to harm (second to implantology), while unnecessary tooth loss is the most common injury followed by nerve damage^24^.

As shown in the results, patient-safety incidents can increase the cost of healthcare substantially. The financial costs associated with patient-harm claims are another key aspect examined in this study. The substantial costs, totalling €16,309,310, highlight the economic burden caused by treatment-related harms in dental care. The reported expenses were mostly related to the repair of an injury and only to a lesser extent include payment for pain and suffering, which is not a pronounced phenomenon in the Danish compensation system. An exception to this is in cases of nerve damage, where compensation for pain and suffering is a significant part of the financial costs. Surgical treatments represent a significant proportion of the costs, followed by endodontic treatments and harms resulting from neglected or delayed diagnosis and initiation of treatment. These findings emphasize the need for preventive measures and early intervention to mitigate patient harm, not only for patient safety reasons but also to reduce the financial impact on both patients and the healthcare system. This should be the goal of future research^23^.

## Future perspectives

The need for increased attention to harm in primary dental care is emphasized^19,20^. Understanding contributing processes can lead to safer systems and diagnostic practices, reducing patient harm. Dentists should prioritize ongoing efforts to identify and mitigate treatment risks, including record keeping, audits, and a safe working culture^25,26^. Implementing prevention strategies and enhancing diagnostic safety improves patient care and reduces financial and emotional costs.

The study offers crucial insights to assist healthcare professionals in directing their efforts towards ensuring safety and reducing the potential risks associated with treatments. Measures include training, guidelines, protocols, and advanced technologies to reduce harm^27,28^. Robust reporting systems should be established to monitor and report harms, utilizing data for trend analysis^29^. Artificial intelligence shows promise in this area. A non-blame culture of safety is fostered by transparency and learning from treatment harm processes. Data on incidents facilitates identifying areas of concern and taking corrective actions, improving patient outcomes.

Future research should explore multiple causes of harm in primary dental care. Retrospective studies use root cause analysis (RCA) to identify contributing factors, improve processes and prevent errors^12,25,30^. Failure mode and effect analysis (FMEA) creates a "risk map" to prevent future harm based on current knowledge (FMEA)^31–33^.

Psychological safety and a positive working environment are crucial for patient safety^34^. Quality and safety are intertwined. Research in primary dental care can draw lessons from the medical field's research on quality and safety^6,35,36^.

## Limitations

While this study provides valuable insights into patient safety incidents in dental care, it is essential to acknowledge its limitations. The data used in this study is derived from the DDCA database, which focuses on treatment-related harms and compensations. This may result in underrepresentation of the full spectrum of patient harm in primary dental care, as incidents that do not lead to compensation claims may not be captured. Additionally, the retrospective nature of the study introduces potential biases and limitations inherent to the analysis of existing data. Moreover, the generalizability of the findings may be limited to the Danish context and healthcare system and to the fact that there might be cases that have not been reported to the scheme since there might be many that are managed “internally”. Different countries may have varying insurance schemes, dental care practices, and reporting systems, which could affect the prevalence and characteristics of patient safety incidents. Therefore, caution should be exercised when extrapolating the findings of this study to other healthcare settings. However, the trends in the data might be global.

To overcome these limitations and further advance the understanding of patient safety in dental care, future research should consider broader data sources, prospective study designs, and multi-center collaborations. These approaches would provide a more comprehensive and representative picture of patient safety incidents, enabling the development of evidence-based strategies and interventions to enhance patient safety.

## Conclusion

More than half of all claims (56%) through the Danish Dental Compensation Act were found to be eligible for compensation. The three most frequent categories leading to patient harm and payment of compensation are "Root canal treatment and post preparation", “lack of timely diagnosis and initiation of treatment” and “surgery”. Damage to the root of the tooth account for more than half of all approved claims (54%) and this is a result of either root canal perforation during endodontic treatment or instrument fracture that led to apical infection and tooth loss. A total of more than €16 m were spent in compensation payments for the studied 5-year period, 41% of which was related to surgery (€6.7 m) and 21% (€3.3 m) to endodontic treatment.

This comprehensive analysis documents that harm permeates all aspects of primary dental care, especially in endodontics and surgery. However, neglect or diagnostic delays contribute to 18% of claims, challenging the notion that harm solely results from direct treatment. Treatment harm inflicts considerable societal costs.

This study offers a comprehensive portrayal of patient safety incidents occurring in various treatment-related harms in dental care. By extracting detailed and standardized information, this study provides the first step in patient-safety research that will ultimately lead to developing evidence-based strategies to prevent such incidents in the future.

### Supplementary Information

Supplementary Figure 1.

Supplementary Figure 2.

## Data Availability

The data underlying this article will be shared on reasonable request to the corresponding author.
