# Supplementary material for: Lessons learnt on patient safety in dentistry through a 5-year nationwide database study on iatrogenic harm
Source: Sci Rep. 2024 May 19;14:11436. doi: 10.1038/s41598-024-62107-x (PMC11102909; doi:10.1038/s41598-024-62107-x)
Supplement: Supplementary file 1 — Supplementary Figure 1. [file 41598_2024_62107_MOESM1_ESM.docx]

**Supplementary Figure 1. Surgical treatment categories leading to harm for the period from 2016-2020 (n=878).**
